# Visceral adipose accumulation increased the risk of hyperuricemia among middle-aged and elderly adults: a population-based study

**DOI:** 10.1186/s12967-019-2074-1

**Published:** 2019-10-10

**Authors:** Xiaolin Huang, Xiaohong Jiang, Long Wang, Lu Chen, Yang Wu, Pei Gao, Fei Hua

**Affiliations:** Department of Endocrine and Metabolic Diseases, The Third Affiliated Hospital of Soochow University, 185 Juqianjie Road, Changzhou, 213000 Jiangsu China

**Keywords:** Hyperuricemia, Visceral adiposity index, Lipid accumulation product, Fatty liver index, Body mass index, Waist circumference, Neck circumference

## Abstract

**Background:**

The role of body fat distribution in uric acid metabolism is still ambiguity. We aimed to investigate the independent contribution of visceral adipose measured by visceral adiposity index and lipid accumulation product and liver fat assessed by fatty liver index to the risk of hyperuricemia.

**Methods:**

We conducted a cross-sectional study involving 1284 participants aged ≥ 40 years old recruited from communities in Zhonglou district, Changzhou. Each participant completed a standard questionnaire, and provided blood samples for biochemical measurements. Visceral adiposity index, fatty liver index and lipid accumulation product were calculated by simple anthropometric and functional parameters. Hyperuricemia was defined as serum uric acid ≥ 420 μmol/l for males and ≥ 360 μmol/l for females.

**Results:**

The prevalence of hyperuricemia was 15.9% and gradually increased across tertiles of adiposity-based indices. The visceral adipose-based measurements (visceral adiposity index, fatty liver index, lipid accumulation product) had better power to discriminate hyperuricemia than body mass index (BMI), waist circumference and neck circumference, and visceral adiposity index exhibited the highest power, with the area under the receiver operating characteristics curve (AUROC) of 0.662 (0.636–0.688). Multivariate logistic regression found 1.49-fold, 2.21-fold and 2.12-fold increased risk of hyperuricemia with 1-unit increment of visceral adiposity index, fatty liver index, and lipid accumulation product, respectively. Compared to tertile 1, the odds ratios of hyperuricemia for the second tertile and the third tertile of visceral adiposity index were 1.57 (1.00–2.50) and 3.11 (1.96–4.94), those of fatty liver index were 1.64 (1.05–2.68) and 3.58 (1.94–6.01), and those of lipid accumulation product were 1.93 (1.19–3.15) and 3.53 (2.05–6.09), respectively. However, no significant associations of BMI, waist circumference and neck circumference with hyperuricemia were observed.

**Conclusions:**

Visceral adipose accumulation increased the risk of hyperuricemia, independently of BMI, waist circumference and neck circumference, among middle-aged and elderly Chinese adults.

## Background

Hyperuricemia, as a consequence of impaired uric acid metabolism, has emerged as a critical public health issue because of its considerable impact on a wide range of clinical morbidity status [1]. Accumulated studies have shown that hyperuricemia not only played a pivotal role in the development of gout, but also significantly associated with hypertension [2], diabetes [3], chronic kidney disease [4] and cardiovascular disease (CVD) [5]. Therefore, the prevention and early detection of hyperuricemia are great important to clinical practice.

Although growing evidence has showed that obesity or excess body fat mass was a risk factor of hyperuricemia [6, 7], the role of body fat distribution in uric acid metabolism is still ambiguity. Previous studies showed that changes of traditional adiposity-based indices, such as body mass index (BMI), waist circumference, neck circumference, were related to the changes of serum uric acid [8–10], demonstrating general obesity or central obesity significantly influenced serum uric acid metabolism. However, BMI is widely used as an index of general obesity, unable to distinguish between central and peripheral fat, subcutaneous and visceral fat, lean mass and fat mass [11]. Waist circumference has been used as a measure of central obesity, but it cannot account for differences in height [12]. Moreover, neck circumference was previously just considered as an anthropometric measure reflected upper body fat distribution [13]. Two recent studies found that visceral fat or liver fat or both were significantly associated with hyperuricemia adjusting for BMI and waist circumference, furthermore, independent of obesity phenotypes [14, 15]. The underlying mechanism may be the excess free fatty acids released from the visceral fat, causing metabolic disorder inflicted by uric acid on the kidney and liver [16]. Drawing a question, does the visceral adipose accumulation have more contribution to the progress of hyperuricemia than adipose deposited in other parts of the body?

Recently, visceral adiposity index, fatty liver index and lipid accumulation product were demonstrated as novel indices of visceral adipose accumulation. Visceral adiposity index calculated by waist circumference, BMI, and triglyceride (TG), high-density lipoprotein cholesterol (HDL-c), was proposed as a novel and accurate indicator of visceral fat distribution and a surrogate marker of adipose tissue dysfunction referred to the quantitative detection by magnetic resonance imaging and computed tomography [17]. Subsequently, several studies reported that visceral adiposity index was a more accurate indicator than other simple anthropometric measures, such as BMI and waist circumference, in predicting incident CVD and diabetes events [18, 19]. In 2005, Kahn [20] proposed lipid accumulation product based on waist circumference and TG, as a practice tool used in the community for reflecting central lipid accumulation. This study also found that lipid accumulation product predict the incidence of CVD better than BMI. Then, more studies further clarified that lipid accumulation product was a better indicator than common obesity indices (BMI, waist circumference, waist-to-hip, waist-to-height ratio) in predicting CVD incidence and other metabolic diseases [21]. Another visceral adiposity indicator, fatty liver index, was based on BMI, waist circumference, TG concentration, and γ-glutamyl transferase (GGT) level. It was also found more diagnostic accuracy for hepatic steatosis than BMI and waist circumference [22]. However, the comparisons between novel visceral adipose-based measures (visceral adiposity index, lipid accumulation product, fatty liver index) and traditional adipose-based indices (BMI, waist circumference, neck circumference) for the risk of hyperuricemia, have not ever been reported.

Hence, the aim of present study was to evaluate and compare the associations of novel visceral adipose-based indices (visceral adiposity index, lipid accumulation product, fatty liver index) with hyperuricemia to other traditional adiposity-based indices (BMI, waist circumference, neck circumference) in middle-aged and elderly adults, contributing to the early diagnosis and therapeutic intervention of hyperuricemia.

## Materials and methods

### Study population

In present study, eligible participants were those who had lived in Zhonglou district more than 6 months, aged 40 years old or above, and without a history of cancer. A total of 1328 participants were enrolled from the Zhonglou district, Changzhou, China, from December 2016 to December 2017. A standard questionnaire for medication information and blood samples collection for biochemical measurements were conducted in each participant. For present analysis, participants were excluded as follows: (1) those with missing data of waist circumference, neck circumference, BMI; (2) those with a treatment of renal disease; (3) those with advanced renal dysfunction (estimated glomerular filtration rate (eGFR) < 30 ml/min/1.73 m^2^). Eventually, 1284 participants included in the present study.

Written informed consent was obtained by each participant and the protocol of present study was approved by Ethics Committee of the Third Affiliated Hospital of Soochow University.

### Data collection

The information on sociodemographic characteristics (marital status, educational level, employment, and family income), lifestyle factors (drinking and smoking habits, and physical activity), as well as medical history (diabetes, hypertension, CVD, the other diseases, and the use of medications) was accessed by trained interviewers through a face-to-face interview using a detailed questionnaire. Those who smoked a cigarette per day or seven cigarettes per week and consumed alcohol at least once a week in the past 6 months were defined as current smokers and current drinkers, respectively. Physical activity at leisure time including the information on intensity, frequency and duration, was evaluated according to the International Physical Activity Questionnaire (IPAQ), categorized as high physical activity or not.

Anthropometric measurements were conducted by trained staffs according to standard protocols. Height and weight were measured with participants barefoot and in light-weight clothes to the nearest 0.1 cm and 0.1 kg, respectively. As the patient exhaled, waist circumference was measured with an inelastic tape positioned between the lowest rib and the top of iliac crest to the nearest 0.1 cm. Neck circumference was measured superior to the thyroid cartilage perpendicular to the long axis of the neck to the nearest 0.1 cm. BMI was calculated as weight in kilograms divided by squared height in meters (kg/m^2^). Blood pressure was measured using an automated electronic device (OMRON Model HEM-752 FUZZY, Omron Company, Dalian, China) at non-dominant arm. After 5-min rest, the measurement was taken three times with 1-min interval. Systolic blood pressure (SBP) and diastolic blood pressure (DBP) in the analysis were defined as the average of three readings.

### Biochemical measurements

Blood samples were collected after at least 10-h overnight fast. Triglyceride (TG), low-density lipoprotein cholesterol (LDL-c), high-density lipoprotein cholesterol (HDL-c), total cholesterol (TC), and GGT, aspartate aminotransferase (AST), alanine aminotransferase (ALT), creatinine, uric acid were measured by an autoanalyser (AU-5800 Chemistry System, Beckman, USA). Fasting plasma glucose (FPG) was measured using the glucose oxidase method with an autoanalyser (AU-5800 Chemistry System, Beckman, USA). According to the Chronic Kidney Disease Epidemiology Collaboration (CKD-EPI), eGFR was calculated as follows [23]: (1) females: Cr ≤ 0.7 mg/dl, eGFR = 144 × (Cr/0.7) − 0.329 × (0.993) age; Cr > 0.7 mg/dl, eGFR = 144 × (Cr/0.7) − 1.209 × (0.993) age. (2) Males: Cr ≤ 0.7 mg/dl, eGFR = 141 × (Cr/0.9) − 0.411 × (0.993) age; Cr > 0.7 mg/dl, eGFR = 141 × (Cr/0.9) − 1.209 × (0.993) age.

### Definitions

#### Hyperuricemia

In the present study, males with serum uric acid ≥ 420 μmol/l and females with serum uric acid ≥ 360 μmol/l were defined as hyperuricemia [24].

#### Visceral adipose-based measurements

1. Visceral adiposity index was calculated as follows [25]: (1) males: visceral adiposity index = [waist circumference (cm)/(39.68 + (1.88 × BMI))] × (TG (mmol/l)/1.03) × (1.31/HDL-c (mmol/l)); (2) females: visceral adiposity index = [waist circumference/(36.58 + (1.89 × BMI))] × (TG/0.81) × (1.52/HDL-c).
2. Fatty liver index was calculated as follows [26]: fatty liver index = (e 0.953 × loge (TG) + 0.139 × BMI + 0.718 × loge (GGT) + 0.053 × waist circumference − 15.745)/(1 + e 0.953 × loge (TG) + 0.139 × BMI + 0.718 × loge (GGT) + 0.053 × waist circumference − 15.745) × 100.
3. Lipid accumulation product was calculated as the following formula [20]: (1) males: lipid accumulation product = (waist circumference (cm) − 65) × TG (mmol/l); (2) females: lipid accumulation product = (waist circumference − 58) × TG.

### Statistical analyses

All statistical analyses of the present study were performed with SAS version 9.3 (SAS Institute Inc, Cary, neck circumference, USA). Continuous variables in the present study were presented as mean ± standard deviation (SD) and medians (interquartile range), while categorical variables were presented as numbers (proportions). Visceral adiposity index, fatty liver index, lipid accumulation product, TG, FPG, ALT, AST and GGT were in skewed distribution and transformed logarithmically. Comparisons between individuals with and without hyperuricemia, were analyzed with *t* test for continuous variables and Chi-square for categorical variables. Linear regression was used to analyze the trend of hyperuricemia across the tertiles of BMI, waist circumference, neck circumference, visceral adiposity index, fatty liver index and lipid accumulation product. Receiver-operating characteristic (ROC) analyses were performed to evaluate the diagnosis ability of adiposity-based indices for hyperuricemia. Comparisons of the area under the receiver operating characteristics curve (AUROC) between visceral adipose-based indices (visceral adiposity index, fatty liver index, lipid accumulation product) and other adiposity-based measurements (BMI, waist circumference, neck circumference) were conducted by using the method described by De Long et al. [27] which was a nonparametric approach using the theory developed for generalized *U*-statistics. Pearson’s correlations of serum uric acid with potential confounding factors for hyperuricemia and stepwise linear regression was further conducted to determine the independents of serum uric acid.

Multivariable logistic regression analyses were used to detect the associations between adiposity-based indices (continuous variables or categorical variables) and the risk of prevalent hyperuricemia. For the serum uric acid levels varies with age and sex, model 1 was adjusted for age, sex. Previous work found that lifestyle is a key contributing factor for the development of hyperuricemia or gout, particularly cigarettes and alcohol consumption and physical activity [28–30]. Hence, model 2 was further adjusted for status of smoking and drinking, physical activity on the basis of model 1. Due to the influence of metabolic status and renal function on the progression of hyperuricemia [31], model 3 was further adjusted for SBP, FPG, TC, LDL-c and eGFR based on model 2. Finally, in order to investigate whether the associations of visceral adipose-based measures with hyperuricemia were independently of the other adiposity-based indices, model 4 was further adjusted for BMI, waist circumference, neck circumference (for analyses about visceral adiposity index, fatty liver index, lipid accumulation product) and visceral adiposity index (for analyses about BMI, waist circumference, neck circumference) based on model 3, respectively.

Two-tailed *P* values less than 0.05 were considered statistically significant in all significance tests.

## Results

### Characteristics of study population

The mean age of study population was 67.8 ± 8.6 years old and the proportion of males was 34.2%. The mean serum uric acid was 311 ± 77 μmol/l and 204 participants (15.9%) were defined as hyperuricemia. Table 1 showed the detailed clinical and biochemical characteristics of individuals with and without hyperuricemia. Participants with hyperuricemia were older, more likely to be females, had higher levels of TG and GGT, but lower levels of DBP, HDL-c and eGFR (all *P* values < 0.05). No difference was detected in status of smoking and drinking, physical activity, SBP, TC, LDL-c, FPG, ALT or AST. Among adiposity-based measurements, levels of BMI, waist circumference, visceral adiposity index, fatty liver index and lipid accumulation product were higher in individuals with hyperuricemia, compared to those without hyperuricemia (all *P* values < 0.05).

**Table 1:** Characteristics of study population

Variables	Total participants	Non-hyperuricemia	Hyperuricemia	*P*
N, %	1284	1080 (84.1)	204 (15.9)	–
Serum uric acid (μmol/l)	311 ± 77	288 ± 57	431 ± 60	< 0.0001
Visceral adiposity index	2.17 (1.41–3.29)	2.08 (1.31–3.08)	2.84 (1.87–4.57)	< 0.0001
Fatty liver index	37.9 (20.8–59.6)	36.2 (19.5–58.1)	48.4 (31.0–70.6)	< 0.0001
Lipid accumulation product	47.7 (29.6–73.0)	45.8 (27.9–68.2)	62.8 (41.0–91.5)	< 0.0001
BMI (kg/m^2^)	25.4 ± 3.5	25.2 ± 3.4	26.1 ± 3.7	0.001
Waist circumference (cm)	89.8 ± 9.6	89.5 ± 9.6	91.4 ± 9.0	0.01
Neck circumference (cm)	35.6 ± 3.5	35.5 ± 3.5	35.8 ± 3.5	0.22
Age (years)	67.8 ± 8.6	67.4 ± 8.5	69.7 ± 8.7	0.0007
Male [n (%)]	439 (34.2)	384 (35.6)	55 (27.0)	0.018
Current smoker [n (%)]	164 (12.8)	144 (13.3)	20 (9.8)	0.17
Current drinker [n (%)]	68 (5.3)	58 (5.4)	10 (4.9)	0.78
High physical activity [n (%)]	751 (58.5)	637 (59.0)	114 (55.9)	0.41
SBP (mmHg)	134 ± 15	134 ± 14	134 ± 15	0.98
DBP (mmHg)	83 ± 9	83 ± 9	82 ± 9	0.04
TG (mmol/l)	1.62 (1.19–2.23)	1.57 (1.14–2.14)	1.92 (1.44–2.86)	< 0.0001
TC (mmol/l)	5.11 ± 0.98	5.11 ± 0.97	5.10 ± 1.06	0.91
LDL-c (mmol/l)	2.70 ± 0.69	2.71 ± 0.69	2.69 ± 0.72	0.79
HDL-c (mmol/l)	1.34 ± 0.30	1.36 ± 0.30	1.23 ± 0.26	< 0.0001
FPG (mmol/l)	5.60 (4.80–6.88)	5.60 (4.80–6.95)	5.58 (4.79–6.61)	0.34
ALT (U/l)	18.0 (14.0–25.0)	18.0 (14.0–25.0)	18.0 (14.0–25.0)	0.56
AST (U/l)	23.0 (19.0–27.0)	22.1 (19.0–27.0)	23.0 (19.0–28.4)	0.23
GGT (U/l)	21.0 (15.0–30.0)	20.0 (15.0–29.0)	23.0 (17.0–32.8)	0.0029
eGFR (ml/min/1.73 m^2^)	80.9 ± 14.2	82.8 ± 12.8	70.5 ± 16.5	< 0.0001

Data were presented as mean ± SD for median (interquartile ranges) for continuous variables, and numbers (proportions) for categorical variables

*P* values were calculated by *t* test for continuous variables and Chi-square test for categorical variables

### Serum uric acid and risk factors

We found that age, SBP, BMI, waist circumference, neck circumference and log-transformed visceral adiposity index, fatty liver index, lipid accumulation product were significantly related with serum uric acid by Pearson correlation. Multivariate stepwise linear regression analysis further revealed that age, neck circumference, log-transformed FPG, visceral adiposity index, fatty liver index, lipid accumulation product were independent factors for serum uric acid (*P* < 0.0001) (Table 2).

**Table 2:** Pearson’s correlation and stepwise regression of determinants of serum uric acid

Variables	r	P*	Standardized *β*	*P* ^#^
Age (years)	0.12	< 0.0001	1.10	< 0.0001
SBP (mmHg)	0.06	0.025	–	
DBP (mmHg)	0.03	0.28	–	
Log (FPG)	0.07	0.07	− 4.67	< 0.0001
TC (mmol/l)	0.22	0.22	–	
LDL-c (mmol/l)	0.39	0.39	–	
BMI (kg/m^2^)	0.20	< 0.0001	–	
Waist circumference (cm)	0.20	< 0.0001	–	
Neck circumference (cm)	0.27	< 0.0001	4.43	< 0.0001
Log (visceral adiposity index)	0.21	< 0.0001	36.3	< 0.0001
Log (fatty liver index)	0.30	< 0.0001	24.6	< 0.0001
Log (lipid accumulation product)	0.25	< 0.0001	− 30.7	0.00025

P* values were calculated by Pearson’s correlation and *P*^#^ values were calculated by multiple stepwise linear regression analysis

### Adiposity-based measurements discriminate the presence of hyperuricemia

We performed ROC analysis to assess the power of adiposity-based measurements to discriminate participants with or without hyperuricemia. Moreover, we compared the AUROC of each visceral adipose-based measurements (visceral adiposity index, fatty liver index, lipid accumulation product) to that of traditional adiposity-based measurements (BMI, waist circumference, neck circumference) (Table 3). The visceral adiposity index exhibited the highest power of discrimination for hyperuricemia, with the AUROC of 0.662 (0.636–0.688), while fatty liver index with AUROC of 0.621 (0.594–0.648), as well as lipid accumulation product with AUROC of 0.645 (0.618–0.671), showed more discriminative power than BMI, waist circumference and neck circumference (all *P* values < 0.05).

**Table 3:** Area under the receiver operating characteristic curve (AUROC) for identifying hyperuricemia with adiposity-based indices

Variables	AUROC	Threshold	Sensitivity	Specificity	*P* ^*#*^
BMI (kg/m^2^)	0.568 (0.540–0.595)	25.2	58.3	53.8	0.0008
Waist circumference (cm)	0.558 (0.530–0.568)	81	89.2	21.4	0.0002
Neck circumference (cm)	0.522 (0.494–0.550)	34	65.2	41.2	< 0.0001
Visceral adiposity index	0.662 (0.636–0.688)*	2.74	55.8	68.2	–
Fatty liver index	0.621 (0.594–0.648)*	30	77.0	41.1	0.04
Lipid accumulation product	0.645 (0.618–0.671)*	71.0	45.6	77.1	0.14

*The AUROC was statistically greater than that of BMI, waist circumference, neck circumference (all *P* values < 0.05). *P*^#^ was calculated by using the method described by De Long. et al. comparing the AUROC of visceral adiposity index with the other adiposity-based indices

### Adiposity-based measurements and the risk of hyperuricemia

**Fig. 1:**
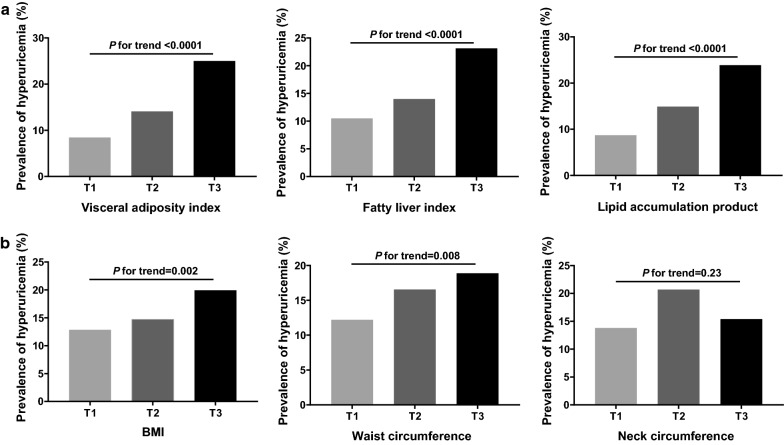
Prevalence of hyperuricemia in different tertiles of adiposity-based indices. **a** Visceral adipose-based indices; **b** other adiposity-based indices. *P* values for trend were calculated by Cochran–Mantel–Haenszel (CMH) method adjusting for age and sex

Figure 1 showed the prevalence of hyperuricemia according to tertiles of adiposity-based measurements. From the lowest tertile to the highest tertile of visceral adiposity index, the prevalence of hyperuricemia was 8.5%, 14.1% and 25% (*P* for trend < 0.0001). According to the increased tertiles of fatty liver index and lipid accumulation product, the prevalence of hyperuricemia was 10.5%, 14.0%, 23.1% and 8.7%, 14.9%, 23.9%, respectively (all *P* values for trend < 0.0001). Likewise, the prevalence of hyperuricemia trended to increase with the elevation of BMI and waist circumference (all *P* values for trend < 0.05), but the same association was not found between neck circumference and prevalent hyperuricemia.

Multivariate logistic regression analyses were used to further assess the associations of adiposity-based measurements, which were performed as continuous variables, with the risk of prevalent hyperuricemia (Table 4). In age- and sex-adjusted logistic regression model, we found that the risk of prevalent hyperuricemia increased with 1-unit increment of visceral adipose-based indices (visceral adiposity index, fatty liver index, lipid accumulation product), as well as other adiposity-based measurements (BMI, waist circumference, neck circumference). After further adjusting for the smoking and drinking habits, SBP, FPG, TC, LDL-c, eGFR, 1.55-fold, 1.04-fold and 1.48-fold increased risk of hyperuricemia were found with 1-unit increment of visceral adiposity index, fatty liver index, lipid accumulation product, respectively. Further adjusting for BMI, waist circumference, neck circumference, the magnitude of associations did little change in visceral adiposity index and significantly increased in fatty liver index and lipid accumulation product. Likewise, adjusting for the smoking and drinking habits, SBP, FPG, TC, LDL-c, eGFR, associations of hyperuricemia with BMI, waist circumference and neck circumference remained significant. However, no significant results were detected further adjusting for log-transformed visceral adiposity index.

**Table 4:** The risk of prevalent hyperuricemia with 1-unit increase of adiposity-based measurements

Variables	OR (95% CI)			
	Model 1	Model 2	Model 3	Model 4
BMI	1.07 (1.03–1.12)	1.07 (1.03–1.12)	1.07 (1.03–1.12)	1.04 (0.99–1.09)
Waist circumference	1.02 (1.00–1.04)	1.02 (1.00–1.04)	1.02 (1.00–1.04)	1.01 (0.99–1.03)
Neck circumference	1.06 (1.01–1.12)	1.06 (1.01–1.12)	1.06 (1.01–1.12)	1.02 (0.96–1.08)
Log (visceral adiposity index)	2.39 (1.86–3.07)	2.40 (1.86–3.09)	2.55 (1.93–3.36)	2.49 (1.87–3.31)
Log (fatty liver index)	2.01 (1.56–2.59)	2.01 (1.55–2.59)	2.04 (1.56–2.66)	3.21 (2.09–4.92)
Log (lipid accumulation product)	2.25 (1.73–2.91)	2.25 (1.73–2.91)	2.48 (1.85–3.22)	3.12 (2.17–4.51)

Model 1: adjusted for age, sex

Model 2: further adjusted for smoking and drinking status, physical activity

Model 3: further adjusted for SBP, FPG, TC, LDL-c, eGFR

Model 4: further adjusted for neck circumference, waist circumference, BMI (for visceral adiposity index, fatty liver index and lipid accumulation product) and visceral adiposity index (for BMI, waist circumference, neck circumference)

Then, the associations of visceral adipose-based indices, which were presented as categorized variables, with risk of prevalent hyperuricemia were showed in Additional file 1: Table S1. According to the increment of visceral adiposity index, the risk of prevalent hyperuricemia gradually increased, after adjusting for age, sex, smoking and drinking status, physical activity, SBP, FPG, TC, LDL-c, eGFR, and BMI, waist circumference, neck circumference. Referred to participants in tertile 1, odds ratios of those in tertile 2 and tertile 3 were 1.57 (1.00–2.50) and 3.11 (1.96–4.94), respectively (*P* for trend < 0.0001). The analogous results were also found in associations of fatty liver index, lipid accumulation product with hyperuricemia.

## Discussion

The present study demonstrated that visceral adipose accumulation is a critical risk factor of prevalent hyperuricemia, independent of various confounding factors and the traditional adiposity-based measurements (BMI, waist circumference, neck circumference) among middle-aged and elderly Chinese adults. With the increment of visceral adiposity index, fatty liver index, lipid accumulation product, the risk of prevalent hyperuricemia gradually increased, whereas no significant relationships existed between BMI, waist circumference, neck circumference and the risk of hyperuricemia. Additionally, findings of the current study clarified that visceral adiposity index is a better indicator for discrimination of hyperuricemia, compared to other visceral adipose-based indices (fatty liver index, lipid accumulation product).

Until now, numerous studies reported associations between obesity and hyperuricemia. However, researches explored the impact of visceral adipose accumulation on risk of prevalent hyperuricemia are few. In the earlier, studies performed by Takahashi, et al. and Matsuura et al. [16, 32] proposed that accumulation of visceral adipose, which was elevated by CT scan, had a great effect on the metabolism of uric acid, which was also stronger than subcutaneous fat or BMI. Nevertheless, the small sample size of these two studies, and participants recruited from outpatient department and obesity patients would limited the generalization of results. Another cross-sectional study enrolled 580 Japanese males [33] and the study conducted in Korea with 699 type 2 diabetes [34] draw the consistent conclusions, the CT-scan visceral fat area, but not the subcutaneous fat area, was positively associated with serum uric acid levels in type 2 diabetes. However, both studies did not take the confounding effect of BMI and lifestyle factors into consideration, and participants of the latter study were restricted to type 2 diabetes patients. Recently, a cross-sectional study conducted in 862 males for medical checkup showed that no matter great amounts of visceral fat or liver fat, which was detected by CT scan, had a dose–response relationship with hyperuricemia, independent of age, lifestyle factors and other adipose depots, yet study population just included males was the main limitation of study design [14]. Results from the other recent study in large Chinese population were partly in line with our study, demonstrated that visceral adiposity index was significantly associated with hyperuricemia in each stratum of obesity phenotypes [15]. In this study, obesity was defined by BMI, but the fat distribution of upper body and central obesity were not taken into consideration.

By including 1284 middle-aged and elderly Chinese adults recruited from communities, we found that visceral fat and liver fat assessed by visceral adiposity index, fatty liver index and lipid accumulation product were crucial risk factors for hyperuricemia adjusting for not only traditional confounders such as age and lifestyle factors but also traditional adiposity-based measurements (BMI, waist circumference, neck circumference). To our knowledge, this is the first large population-based study to clarify not only the association of visceral adipose with hyperuricemia but also that of liver fat accumulation with hyperuricemia, compared to the traditional simple anthropometric measurements including BMI, waist circumference, and neck circumference.

In the present study, visceral adipose accumulation was presented by visceral adiposity index, fatty liver index and lipid accumulation product. The gold standard for determining the extent of visceral fat area are magnetic resonance imaging (MRI) and computed tomography [35]. Due to time-consuming, expense and radiologic hazard, MRI and CT could not be applied to large epidemiological studies. Visceral adiposity index proposed by Amato et al. based on anthropometric (waist circumference, BMI) and metabolic variables (TG, HDL-c) and lipid accumulation product calculated by waist circumference and TG were used for pathogenic fat depot of visceral adipose tissue instead. As for visceral adiposity index, the accuracy and generalizability to various ethics for diagnosis of visceral adipose had been ever confirmed [17, 36]. Previous studies revealed that visceral adiposity index was an elevated risk factor of cardiovascular diseases [37], hypertension [38] and renal dysfunction [39]. Our findings extended the clinical function of visceral adiposity index into a predictor of prevalent hyperuricemia, enhancing the application of visceral adiposity index to screen metabolic and vascular diseases in large epidemiological studies. Alternatively, lipid accumulation product, which had offered a well-known relation to the quantity of visceral fat, independently correlated with metabolic disorders and incident cardiovascular events [40–42]. The positive association of lipid accumulation product and risk of hyperuricemia in the current study further elaborated its influence on the uric acid metabolism. Fatty liver index, the algorithm based on BMI, waist circumference, TG and GGT, has been validated as practical, reliable and economic technique to diagnose fatty liver and quantify hepatic steatosis [22]. Currently, fatty liver was reported as a risk factor proceeded the progression of hyperuricemia. Our study found that elevated fatty liver index independently increased the risk of prevalent hyperuricemia, which was consistent with previous findings.

The major strength of the present study was the large sample size, randomly selected from communities with limited sampling bias. In addition, we evaluated and compared the association of novel visceral adipose-based indices (visceral adiposity index, fatty liver index, lipid accumulation product) with hyperuricemia to other traditional adiposity-based indices (BMI, waist circumference, neck circumference), extended the previous data. However, several limitations of our study should be acknowledged. First, since the cross-sectional nature of our study, no causal inference can be established. Moreover, Neyman bias may existed in this cross-sectional study. Participants with prevalent hyperuricemia might have changed their lifestyle, and then reduced BMI and waist circumference, or visceral fat mass. The magnitude of association between visceral adipose distribution and hyperuricemia would be weaken, resulting in more possibility of type II error. Hence, further prospective studies are warranted to interpret the role of visceral adipose accumulation on the progression of serum uric acid metabolism and hyperuricemia. Second, as including the middle-aged and elderly Chinese adults in the present study, it should be cautious to generalize results to other ethnic groups. However, the validations of visceral adiposity index, fatty liver index and lipid accumulation product for assessing obesity or visceral adipose or liver fat in Chinese population had been performed in previous studies [22, 43, 44], so the study design and conclusions drawn from our present study were creditable. Thirdly, the confounding factor urate-lowering therapy was not adjusted in the present study. According to previous studies, the treatment of asymptomatic hyperuricemia was controversial in clinical practice [45], and the rate of adherence to urate-lowering therapy was only 9.6% in Chinese gout patients [46], so the bias of urate-lowering therapy was slight in our study. Finally, recent prospective epidemiological studies supported that dietary factors, such as purine-rich foods, dairy products and soy foods, were responsible for the development of hyperuricemia and gout [47–49]. Nevertheless, the dietary information was not collected in our study, and it would be planned to acquire in the follow-up study using food frequency questionnaire.

## Conclusions

In conclusion, among middle-aged and elderly Chinese population, more visceral adipose accumulation is association with increased risk of hyperuricemia, regardless of BMI, waist circumference and neck circumference. Furthermore, visceral adiposity index showed the highest power of discriminating hyperuricemia among visceral adipose-based indices. Further longitudinal studies are warranted to verify our findings in external populations.

## Supplementary information

**Additional file 1: Table S1.** The risk of prevalent hyperuricemia according to the tertiles of visceral adipose-based measures.

## Data Availability

The datasets in the current study arised from a dataset of Department of Endocrinology, the Third Affiliated Hospital of Soochow University, are not publicly available due to security consideration, but are available from the corresponding author on reasonable request.

## Abbreviations

AUROC: area under the receiver operating characteristic curve
ALT: alanine aminotransferase
AST: aspartate aminotransferase
BMI: body mass index
CI: confidence interval
CKD-EPI: the Chronic Kidney Disease Epidemiology Collaboration
CVD: cardiovascular disease
DBP: diastolic blood pressure
eGFR: estimated glomerular filtration rate
FPG: fasting plasma glucose
GGT: gamma-glutamyl-transferase
HDL-c: high-density lipoprotein cholesterol
IPAQ: the International Physical Activity Questionnaire
LDL-c: low-density lipoprotein cholesterol
OR: odds ratio
SD: standard deviation
SBP: systolic blood pressure
TG: triglyceride
TC: total cholesterol
