# Supplementary material for: Visceral adipose accumulation increased the risk of hyperuricemia among middle-aged and elderly adults: a population-based study
Source: J Transl Med. 2019 Oct 10;17:341. doi: 10.1186/s12967-019-2074-1 (PMC6785935; doi:10.1186/s12967-019-2074-1)
Supplement: Supplementary file 1 — Additional file 1: Table S1. The risk of prevalent hyperuricemia according to the tertiles of visceral adipose-based measures. [file 12967_2019_2074_MOESM1_ESM.docx]

| **Table S1. The risk of prevalent hyperuricemia according to the tertiles of** **visceral adipose-based measures** | | | | |
| --- | --- | --- | --- | --- |
|  | **OR (95% CI)** | | | |
|  | **Tertile 1** | **Tertile 2** | **Tertile 3** | ***P* for trend** |
| **Visceral adiposity index** | | | | |
| Model 1 | 1.00 (Ref) | 1.68 (1.07-2.65) | 3.34 (2.16-5.16) | <0.0001 |
| Model 2 | 1.00 (Ref) | 1.69 (1.08-2.67) | 3.38 (2.18-5.26) | <0.0001 |
| Model 3 | 1.00 (Ref) | 1.65 (1.04-2.83) | 3.29 (2.11-5.13) | <0.0001 |
| Model 4 | 1.00 (Ref) | 1.57 (1.00-2.50) | 3.11 (1.96-4.94) | <0.0001 |
| **Fatty liver index** | | | | |
| Model 1 | 1.00 (Ref) | 1.40 (0.91-2.17) | 2.70 (1.80-4.04) | <0.0001 |
| Model 2 | 1.00 (Ref) | 1.40 (0.91-2.16) | 2.70 (1.80-4.04) | <0.0001 |
| Model 3 | 1.00 (Ref) | 1.44 (0.93-2.25) | 2.70 (1.77-4.11) | <0.0001 |
| Model 4 | 1.00 (Ref) | 1.64 (1.05-2.68) | 3.58 (1.94-6.01) | <0.0001 |
| **Lipid accumulation product** | | | | |
| Model 1 | 1.00 (Ref) | 1.80 (1.15-2.80) | 3.00 (1.96-4.58) | <0.0001 |
| Model 2 | 1.00 (Ref) | 1.79 (1.15-2.80) | 3.00 (1.96-4.58) | <0.0001 |
| Model 3 | 1.00 (Ref) | 1.80 (1.15-2.84) | 3.06 (1.97-4.75) | <0.0001 |
| Model 4 | 1.00 (Ref) | 1.93 (1.19-3.15) | 3.53 (2.05-6.09) | <0.0001 |
| Model 1: Adjusted for age, sex; | | | | |
| Model 2: Further adjusted for smoking and drinking status, physical activity; | | | | |
| Model 3: Further adjusted for SBP, FPG, TC, LDL-c, eGFR | | | | |
| Model 4: Further adjusted for neck circumference, waist circumference, BMI | | | | |
